# Strain induced fragility transition in metallic glass

**DOI:** 10.1038/ncomms8179

**Published:** 2015-05-18

**Authors:** Hai-Bin Yu, Ranko Richert, Robert Maaß, Konrad Samwer

**Affiliations:** 1 Physikalisches Institut, Universität Göttingen, D-37077 Göttingen, Germany; 2 Department of Chemistry and Biochemistry, Arizona State University, Tempe, Arizona 85287, USA; 3 Institute for Materials Physics, University of Göttingen, D-37077 Göttingen, Germany

## Abstract

Relaxation dynamics are the central topic in glassy physics. Recently, there is an emerging view that mechanical strain plays a similar role as temperature in altering the relaxation dynamics. Here, we report that mechanical strain in a model metallic glass modulates the relaxation dynamics in unexpected ways. We find that a large strain amplitude makes a fragile liquid become stronger, reduces dynamical heterogeneity at the glass transition and broadens the loss spectra asymmetrically, in addition to speeding up the relaxation dynamics. These findings demonstrate the distinctive roles of strain compared with temperature on the relaxation dynamics and indicate that dynamical heterogeneity inherently relates to the fragility of glass-forming materials.

## Abstract (web summary)

The equivalency of stress and temperature as driving force for the relaxation in metallic glasses is widely accepted. Here, Yu *et al.* examine this assumption in simulations and find that stress induces a fragile-to-strong transition in addition to accelerated relaxation dynamics as temperature does.

## Graphical abstract

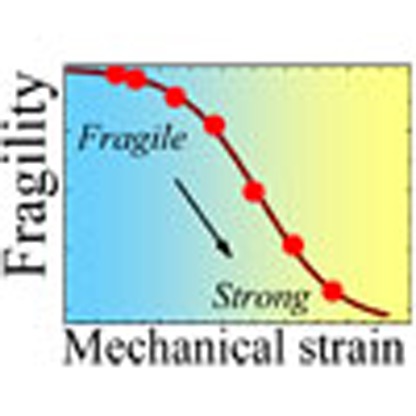

Glasses and supercooled liquids feature diverse and complex relaxation dynamics^1^,^2^,^3^,^4^,^5^,^6^,^7^,^8^. The most prominent relaxation mode is the so-called primary (α-) relaxation, which signals a nonequilibrium transition from a solid-like state to a viscous liquid-like state, characterized solely by the change of dynamics^1^,^2^,^3^,^4^. Recently, it has been recognized that mechanical stress (or strain) plays a similar role as temperature in the dynamics of the α relaxation in many kinds of glass-forming materials^9^, including colloidal glasses^9^,^10^,^11^, granular materials^12^ and metallic glasses (MGs)^13^,^14^,^15^,^16^. Initially, Liu and Nagel^9^ introduced the concept of a jamming diagram to unify the roles of stress, temperature and density in colloidal glasses. The pursuits of this view have also led to some scaling laws in different types of glasses to rationalize the roles of temperature, strain/stress and other variables^13^,^17^. Quantitatively, such jamming diagrams and scaling laws, however, depend on the intrinsic properties of glass-forming materials such as the interaction potentials^9^,^18^, and thus cannot be generalized across different types of glasses.

In the field of MGs, such a view and its equivalent idea that ‘stress/strain-driven glass transitions' have particular significance as they have been taken as an essential ingredient to understand a number of crucial issues, such as the mechanical properties (strength and ductility)^19^,^20^,^21^, mechanisms of plastic deformation^22^,^23^, as well as the origins of shear-banding and serrated flow^24^,^25^,^26^. Despite its importance, we note that there is still no direct characterization of the dynamics of the α relaxation under stress/strain for MGs. Previous results were based on either apparent viscosity or structural perspectives^13^,^14^. Partly, the difficulty stems from the fact that conventional mechanical spectroscopy that probes relaxation dynamics of MGs is not applicable to nonlinear deformation regime where large stress and strain are required^7^,^8^. Therefore, the basic question whether temperature and stress indeed have similar or different role in relaxation dynamics of MGs is still not clear.

In this work, we address this issue by studying the relaxation spectra of a model MG in the parameter space of temperature, frequency and strain amplitude, via a recently proposed molecular dynamics simulation of dynamical mechanical spectroscopy (MD-DMS)^27^ together with structural analysis. We find that mechanical strain not only accelerates the relaxation dynamics as previously assumed but also alters it in unexpected ways: a fragile MG gradually becomes a strong one under increasing strain, together with a phenomenon of broadening the peak of α relaxation on the loss spectra only for the low-temperature side (while the high-temperature side remains almost unchanged). Structurally, these processes are accompanied by a suppression of dynamical heterogeneity at the glass transition, demonstrating the key role of dynamical heterogeneity in controlling the fragility of glass-forming materials.

## Results

### Relaxation spectra

The details of our model system and the protocol of MD-DMS are given in Methods. Briefly, at a temperature *T*, we apply a sinusoidal strain *ɛ*(*t*)=*ɛ*_A_ sin(2*πt*/*t*_ω_), with a period *t*_ω_ (related to frequency *f*=1/*t*_ω_) and a strain amplitude *ɛ*_A_, along the *x* direction of a model Cu_65_Zr_35_ MG and the resulting stress *σ*(*t*) is measured. To study the strain effects on glassy dynamics, we intentionally vary *ɛ*_A_ from linear (elastic) to nonlinear (plastic) deformation regimes. For simplicity, this work focuses on the first Fourier component of the response stress. High-order effects due to nonlinear response will be discussed in a later work. The storage (*E′*) and loss (*E′′*) moduli are calculated as functions of *T*, *t*_ω_ and *ɛ*_A_.

**Figure 1:**
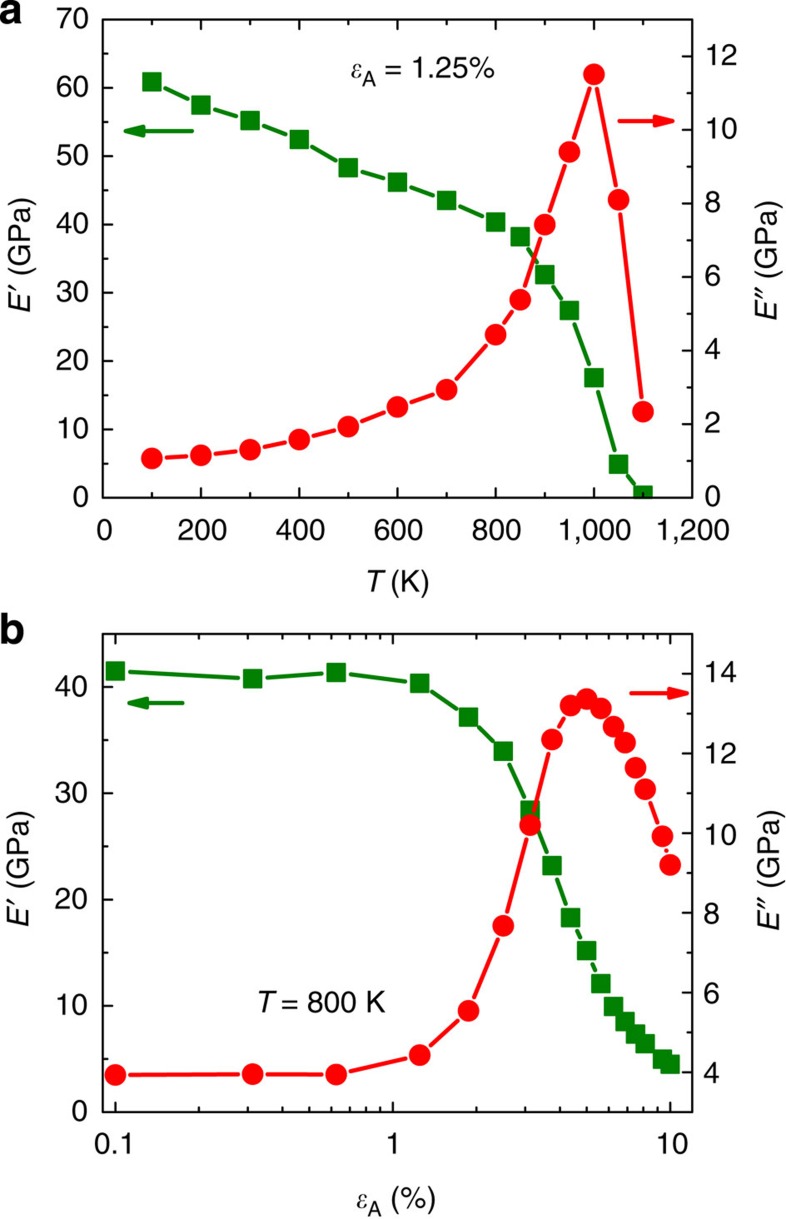
Storage (*E′*) and loss (*E′′*) moduli from MD-DMS. (**a**) E′ and E″ as a function of temperature *T* with a strain amplitude *ɛ*_A_=1.25% (**b**) as a function of *ɛ*_A_ with *T*=800 K. The period is *t*_ω_=1,000 ps.

Figure 1a shows an example of *E′* and *E′′* as a function of *T* for a selected combination of *ɛ*_A_=1.25% and *t*_ω_=1,000 ps (or *f*=1 GHz). They exhibit the typical features of an α relaxation that is consistent with experimental DMS^7^,^8^, that is, a sudden drop of *E′* as well as an asymmetrical peak of *E′′* around *T*_α_=1,000 K (the temperature corresponding to the α relaxation time *τ*_α_=*t*_ω_), that signal the transition from a glassy state to a supercooled liquid state. Figure 1b shows *E′* and *E′′* for a fixed temperature of *T*=800 K as a function of *ɛ*_A_. One can see that for *ɛ*_A_<2%, the values of *E′* are high, while *E′′* are low, and both are nearly independent of *ɛ*_A_, indicating that the model MG is in a glassy state and responses linearly to the external mechanical oscillations. However, with further increase in *ɛ*_A_, *E′* decreases rapidly in a sigmoidal manner, while *E′′* first increases and then decreases, with a peak around *ɛ*_A_=5%. These features, phenomenally similar to Fig. 1a, indicate that the model MG enters into a liquid-like state driven by mechanical strain, that is, the α relaxation takes place at *T*=800 K under *ɛ*_A_=5%, which is 200 K lower than for the case of *ɛ*_A_=1.25%, as shown in Fig. 1a.

**Figure 2:**
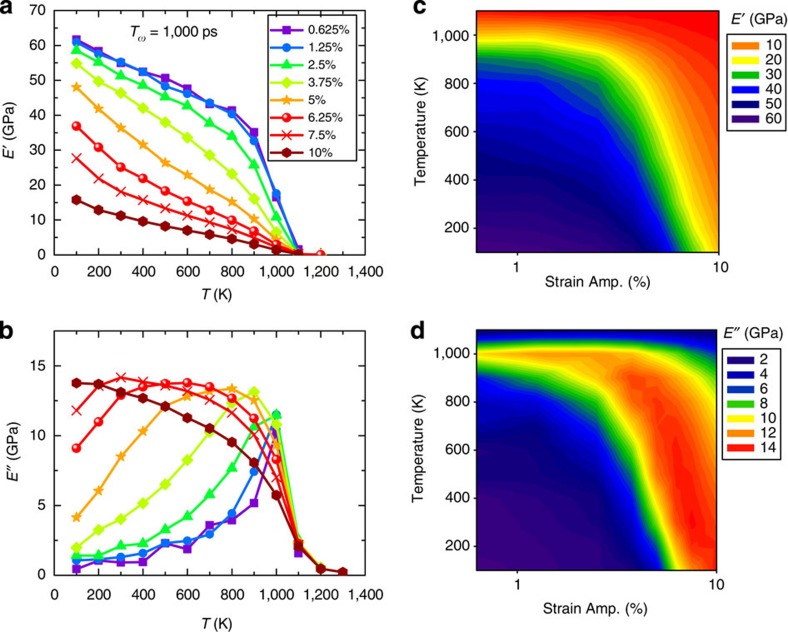
Temperature and strain amplitude dependent modulus. (**a**) *E′* and (**b**) *E′′* as a function of *T* for different strain amplitude levels, *ɛ*_A_. (**c**) *E′* and (**d**) *E′′* contour plots as 2D function of *T* and *ɛ*_A_. The period is *t*_ω_=1,000 ps.

Figure 2a,b shows the *T*-dependent curves for *E′* and *E′′*, respectively, for various values of *ɛ*_A_ at *t*_ω_=1,000 ps. For a better view, Fig. 2c,d recasts the same data in terms of contour plots as two-dimensional (2D) functions of *T* and *ɛ*_A_. The values of *ɛ*_A_ range from 0.625 to 10%, covering both elastic and plastic deformation regimes of the model MG. First, Fig. 2a,b shows a pronounced nonlinear effect on the magnitudes of *E′* and *E′′*, which agrees with Fig. 1b. Second, Fig. 2c,d indicates that the α relaxation can be activated either by *T* or *ɛ*_A_ or a combination of both, and the α relaxation can take place at any temperature depending on *ɛ*_A_. For instance, although *T*_α_=1,000 K in the linear response regime, it is reduced to a temperature as low as 500 K when *ɛ*_A_ approaches 6.25%. For *ɛ*_A_≥10%, we even find that the model MG always behaves like in a liquid-like state at any *T* in our MD-DMS, which means *T*_α_ is practically 0 K in that case. Overall, these results provide convincing evidence that strain can accelerate the relaxation dynamics and support the notion of a mechanically driven liquid–glass transition^13^. Third, we note that Fig. 2b reveals one intriguing feature of nonlinear effects on the first-order mechanical spectra: the *E′′* peak of the α relaxation broadens substantially in the left (low *T*) side, while the right (high *T*) side of it is almost unchanged. This feature implies that strain in the nonlinear regime alters the relaxation dynamics in a distinctive way compared with temperature: if mechanical strain behaves similarly as enhanced effective temperature, then the spectra of *E′′* will just shift to low *T* side as a whole, keeping the shape almost unchanged, and the left-side broadening of *E′′* peak could not be observed. We note that a signature of similar (but much smaller) nonlinear effects is observed in experimental dielectric spectra of organic glasses, which reveal that large amplitudes of electric fields modify only the high-frequency side of the dielectric spectra of the α relaxation during a constant *T* measurement^28^,^29^.

### Relaxation dynamics and fragility

**Figure 3:**
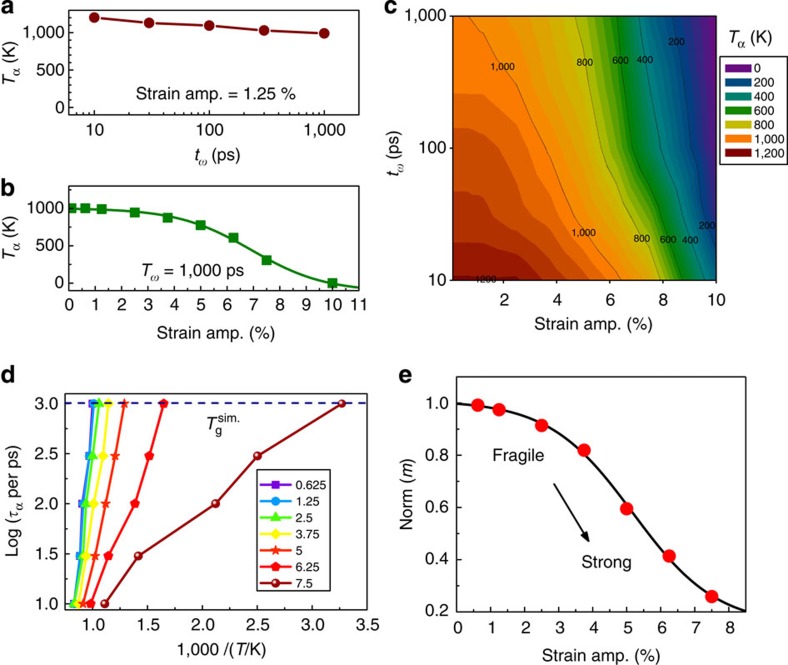
Temperature and strain amplitude dependent α relaxations. (**a**) The primary relaxation temperature *T*_α_ as a function of period *t*_ω_ for *ɛ*_A_=1.25%. (**b**) *T*_α_ as a function of *ɛ*_A_ for *t*_ω_=1,000 ps. (**c**) *T*_α_ as a 2D function of *t*_ω_ and *ɛ*_A_. (**d**) The α relaxation dynamics as a function of 1,000/*T* for different *ɛ*_A_ as indicated in percentage. (**e**) The normalized fragility index *m* as a function of *ɛ*_A_.

Next, we focus on *T*_α_, determined from the peak temperature of *E′′* as a direct indicator of relaxation dynamics. Figure 3a,b shows the values of *T*_α_ as a function of *t*_ω_ and *ɛ*_A_, respectively. One can see that *T*_α_ decreases from 1,200 to 1,000 K as *t*_ω_ increases from 10 to 1,000 ps for *ɛ*_A_=1.25% (Fig. 3a, frequency effects), while it decreases from 1,000 K to almost below 50 K in the nonlinear response regime from *ɛ*_A_=3 to 10% at a fixed *t*_ω_=1,000 ps (Fig. 3b, strain effects). This suggests that mechanical strain is more effective in modifying the relaxation dynamics than temperature within the capacity of MD simulations, since MD is usually limited by timescale; however, the mechanical strain can be changed readily. Figure 3c summarizes how *T*_α_ changes as a 2D function of *ɛ*_A_ and *t*_ω_. A general trend is that *T*_α_ decreases with *ɛ*_A_ for any *t*_ω_ in the nonlinear response regime. However, the magnitude of the decrease in *T*_α_ is larger for higher *t*_ω_. For example, for *t*_ω_=1,000 ps, *T*_α_ decreases from 1,000 K at *ɛ*_A_<2.5% to 350 K at *ɛ*_A_=7.5%, while for *t*_ω_=10 ps *T*_α_ decreases only from 1,200 to 1,000 K with the same *ɛ*_A_.

To quantify the effects of strain on relaxation dynamics, Fig. 3d plots the relaxation time as a function of inverse temperature. One can observe that strain modifies the relaxation dynamics in a nontrivial way. Specifically, the effects of stress are more pronounced at low temperatures than at high temperatures. Thus, the relaxation time is strongly reduced at low temperatures but less at high temperatures. In this connection, it would be useful to consider the concept of fragility^30^,^31^,^32^, quantified by *m*=dlog(*τ*_α_)/d(*T*_*g*_*/T*), that characterizes how rapidly the dynamics of a material slow down as it is cooled towards the glass transition. Liquids with large *m* are called fragile, while those with small *m* are considered strong. In our simulations, the glass transition temperature is defined as *T*_g_^sim^≡*T*_α_ (*t*_ω_=1,000 ps) as shown in Fig. 3d, which for those in the linear response regime agree with the temperature at which the slope of a volume–*T* curve would change. The resulting *m* values are shown in Fig. 3e and are normalized to low-strain-limit fragility, approximated by *m* at *ɛ*_A_=0.625%. We find that *m* is nearly independent of *ɛ*_A_ in the linear response regime as expected, while, intriguingly, it shows pronounced dependence on *ɛ*_A_ in the nonlinear response regime and decreases to ∼1/4 of its linear response value when *ɛ*_A_ reaches 7.5%. Such a large reduction of *m* is nontrivial, as *m* is known to be insensitive to many external variables and is often considered an intrinsic property of glass-forming materials^33^. For example, *m* is only weakly dependent on hydrostatic pressures^34^. In addition, we note that the reduction of *m* underpins the broadening of the spectra of *E′′* in Fig. 2b, as it is known that materials with a higher fragility have a relatively narrow glass transition temperature range, while the stronger materials (those with lower fragility) have a wide glass transition temperature range^32^.

## Discussion

**Figure 4:**
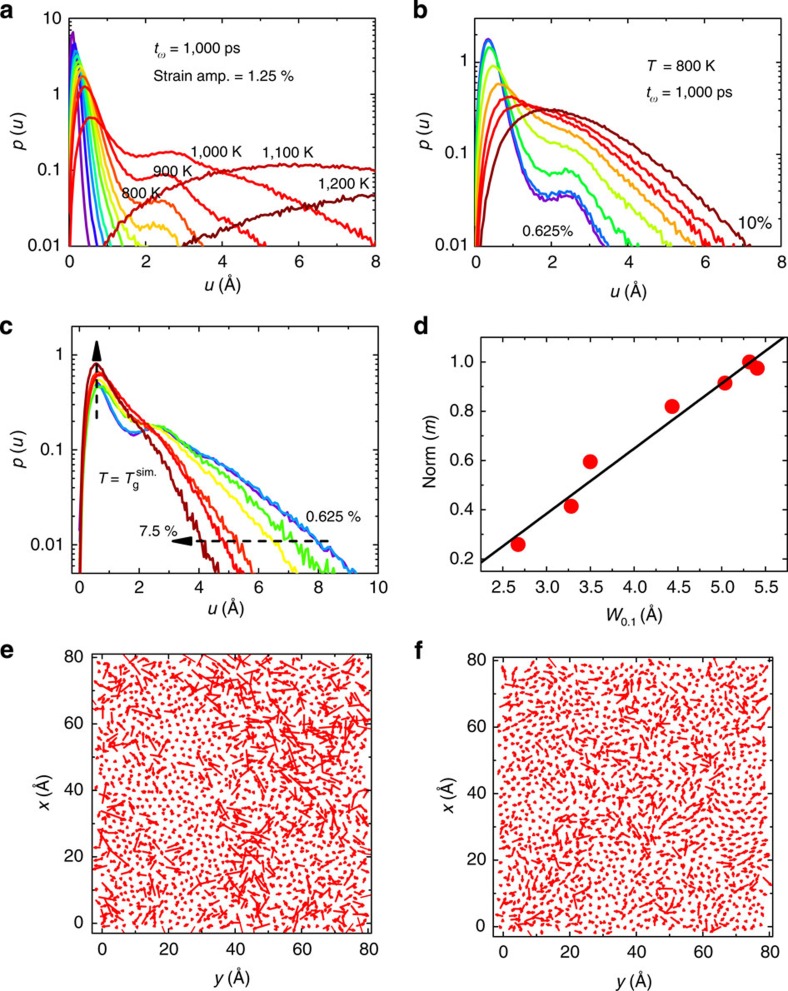
Structural analysis on the basis of the mean square atomic jump distance *u*. (**a**) Probability density *p*(*u*) for different *T* at *t*_ω_*=*1,000 ps and *ɛ*_A_=1.25%, temperature ranges from 100K to 1,200K for every 100K from left to right. (**b**) *p*(*u*) for different *ɛ*_A_ at *T=*800 K and *t*_ω_*=*1,000 ps, the strain amplitudes are 0.625%, 1.25%, 2.5%, 3.75%, 5.0%, 6.25%, 7.5% and 10% from left to right. (**c**) *p*(*u*) at the simulation glass transition temperature *T*_g_^sim^=*T*_α_ (*t*_ω_=1,000 ps) for different *ɛ*_A_ of 0.626%, 1.25%, 2.5%, 3.75%, 5.0%, 6.25% and 7.5% from right to left. (**d**) Relation between normalized fragility *m* and the width of *p*(*u*) at 1/10 maximum *W*_0.1_. (**e**,**f**) 2D *xy* slices (20 Å<*z*≤24 Å) of the vector field of *u* at *T*_g_^sim^ for *ɛ*_A_=1.25% and 7.5%, respectively.

To further study the nonlinear strain effects on the relaxation dynamics and to understand why *m* is dramatically reduced in the nonlinear regime, we conducted a structural analysis and calculated the mean square atomic jump distance *u* for each atom during a time interval of Δ*t*=*t*_ω_ for all the combinations of *T*, *t*_ω_ and *ɛ*_A_. This choice of Δ*t* is meant to avoid atomic displacements due to the overall deformations applied by the MD-DMS. Figure 4a,b shows the resulting probability density function *p*(*u*) for different *T* (where *ɛ*_A_=1.25% and *t*_ω_=1,000 ps remain fixed) and different *ɛ*_A_ (*T*=800 K, *t*_ω_=1,000 ps), respectively. The peak position *u*_*p*_ and the width of *p*(*u*) represents the most probable atomic jump distance of all the atoms and the jump distance dispersion, respectively. The latter quantity is associated with dynamical heterogeneity. From Fig. 4a,b, one can see that both mechanical strain and temperature can increase *u*_*p*_ and broaden the distribution of *p*(*u*), which provides structural evidence for the strain-accelerated dynamics as outlined in Figs 1 and 2. Figure 4c plots *p*(*u*) at *T*=*T*_g_^sim^*=T*_α_ (*t*_ω_=1,000 ps) for different *ɛ*_A_. Interestingly, we find at *T*_g_^sim^ that mechanical strain can reduce substantially the distribution of *p*(*u*), a signature of suppressing the dynamical heterogeneity substantially. Such a behaviour is consistent with some recent simulations and experiments, which reveal that in the presence of mechanical deformation, the dynamics of supercooled liquids are more homogenous, as the deformation reduces the correlation length and the lifetime of dynamical heterogeneity^35^,^36^. Figure 4d plots *m* as a function of *W*_0.1_, where *W*_0.1_ is defined as the width of *p*(*u*) at 1/10 of the maximum as an indicator of dynamical heterogeneity. Remarkably, we observe a strong correlation between *m* and *W*_0.1_, even where the MD-DMS covers both the linear and nonlinear response regimes. Such a correlation clearly suggests that dynamical heterogeneity is a key feature that relates to fragility of glass-forming materials. Figure 4e,f presents two typical slices of 2D vector fields of *u* at *T*=*T*_g_^sim^ for *ɛ*_A_=1.25% and 7.5%, respectively. One can see that the *u*-field with *ɛ*_A_=1.25% (Fig. 4e) is more heterogeneous than the one with *ɛ*_A_=7.5%, corroborating that deformation reduces dynamical heterogeneity in terms of its correlation lengths at the glass transition *T*_g_^sim^.

The results discussed above not only demonstrate that high strain amplitudes can accelerate dynamics, as does an increase in temperature, but also that the value of *τ*_α_ alone does not specify the entire dynamics of the MG system. In other words, two states with identical α relaxation time (one reached by low *T* and high *ɛ*_A_, another reached by high *T* and low *ɛ*_A_) will not coincide regarding other aspects of the dynamics, such as fragility and dynamic heterogeneity. Our explanation is as follows: nonlinear response is reached by strain amplitudes for which the mechanical energy involved per period, ∝*σ*_A_*ɛ*_A_, becomes comparable to or even large than *k*_B_*T*. In a potential energy landscape (PEL) picture^37^,^38^, higher temperature assists activation and modifies the population within a given landscape, whereas high values of *ɛ*_A_ are equivalent to tilting the PEL, which affects low and high barriers in different ways.

In summary, we show that mechanical strain not only accelerates the relaxation dynamics of a model MG, but also modifies it in unique ways. In particular, a fragile glass former becomes stronger under mechanical strain, and the peak of the α relaxation broadens asymmetrically. Structurally, dynamical heterogeneity at the glass transition is gradually suppressed with increasing strain amplitude. These findings emphasize the distinctive roles of strain compared with temperature on the relaxation dynamics, and provide microscopic insights to the concept of fragility as well.

## Methods

### Simulation details and samples

An open source LAMMPS package^39^ was used for the MD simulations. The model system contains *N*=32,000 atoms with the composition Cu_65_Zr_35_, and the constituting atoms are interacting via an embedded atom method potential^40^. For the sample preparations, the system was melted and equilibrated at *T*=3,000 K, and then cooled down to *T*=100 K with a cooling rate of 10^12 ^K s^−1^, during which the cell sizes were adjusted to give zero pressure within the constant number, pressure and temperature (NPT) ensemble. Periodic boundary conditions were applied for all the calculations.

### Simulation of dynamical mechanical spectroscopy

As in the case of experimental DMS, we apply a sinusoidal strain *ɛ*(*t*)=*ɛ*_A_ sin(2*πt*/*t*_ω_) along the *x* direction of the model MG, where *t*_ω_ is the period and is selected as 10, 30, 100, 300 and 1,000 ps, while *ɛ*_A_ is selected as 0.625, 1.25, 2.5, 3.75, 5.0, 7.5 and 10% in this work. For each MD-DMS, 10 full cycles were used, that is, *t* in the range (0, 10*t*_ω_). We fitted the resulting stress as: *σ*(*t*)=*σ*_0_+*σ*_A_ sin(2*πt*/*t*_ω_+*δ*) where *σ*_0_ is a constant term and usually small (*σ*_0_<0.1*σ*_A_ in the glassy state), *δ* the phase difference between stress and strain. Storage (*E′*) and loss (*E′′*) moduli are calculated as *E*′=*σ*_A_/*ɛ*_A_ cos(*δ*) and *E*′′=*σ*_A_/*ɛ*_A_ sin(*δ*), respectively. The MD-DMS was carried out during the cooling processes of the sample preparations, and constant number, volume and temperature (NVT) ensemble was applied during the cyclic deformations. No shear-band formation was observed during the MD-DMS simulations across the entire strain and temperature ranges.

### Jump distance probability density functions *p*(*u*)

The probability density function *p*(*u*) is defined as 
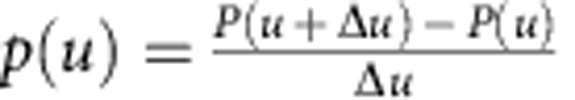
, where *P*(*u*) is the distribution that quantifies the probability of finding *X*≤*u*. Here we employ Δ*u*=0.01 Å for all the calculations. Note that the probability density is normalized according to 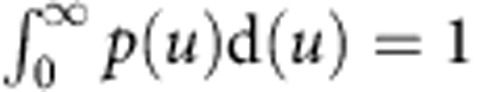.

## Additional information

**How to cite this article:** Yu, H.-B. *et al.* Strain induced fragility transition in metallic glass. *Nat. Commun.* 6:7179 doi: 10.1038/ncomms8179 (2015).
